# Evaluation of functional attributes and storage stability of novel juice blends from baobab, pineapple, and black-plum fruits

**DOI:** 10.1016/j.heliyon.2022.e09340

**Published:** 2022-04-28

**Authors:** Tawakalt O. Adedokun, Athanasia Matemu, Otmar Höglinger, Erasto Mlyuka, Akinbode Adedeji

**Affiliations:** a Department of Food Biotechnology and Nutritional Sciences, Nelson Mandela African Institution of Science and Technology (NM-AIST), P.O. Box 447, Arusha, Tanzania; b Department of Sustainable Agriculture, Biodiversity and Ecosystems Management, Nelson Mandela African Institution of Science and Technology, P.O. Box 447, Arusha, Tanzania; c Department of Food Technology and Nutrition, University of Applied Sciences, Upper Austria; d Department of Biosystemss and Agricultural Engineering University of Kentucky, USA

**Keywords:** Underutilized fruits, Functional beverage, Baobab, Black-plum, Pineapple, Novel juice blend

## Abstract

Several under-utilized tropical fruits have exceptional micronutrients and phytochemical composition with the potential to contribute to the nutrition of people and also enhance food security. This study was carried out to evaluate the quality attributes and storage stability of juice blends of baobab (*Adansonia digitata*), pineapple (*Ananas comosus*), and black-plum (*Syzygium cumini)* fruits for use as functional beverages. Juice blends were analyzed for physicochemical, antioxidant, and sensory properties. Mineral compositions and storage stability of the pasteurized juice blends at 4 °C for 4 weeks were also investigated. Results showed that the vitamin C contents of individual juices synergistically contributed to the high values observed in the blends (317.45–414.51 mg/L). Juice blends of baobab, pineapple, and black-plum fruits are good sources of calcium (57–153 mg/L), magnesium (71–130 mg/L) and antioxidants (ascorbic acid, total polyphenol contents (65–104 mg GAE/100 mL), scavenging ability (105.97–359.71 μmol TE/100 mL), and reducing potential (1376–1829 μMFe^2+^ L) the consumption of which will promote human health. Blending enhanced the sensory qualities of the individual juices with improved taste and consumer acceptability. The juice blends kept well for two weeks at 4 °C though the color becomes less intense at the end of the storage period. These findings suggest that baobab-fruit pulp, pineapple, and black-plum fruits can be major ingredients in producing a consumer acceptable anti-oxidant rich functional beverage for optimal benefits to consumers.

## Highlights

• Fruit juice blends were made from pineapples, baobab, and black-plum fruits.
• The blends are rich sources of essential micronutrients and antioxidants.
• Blending improved juice antioxidant capacity and consumer acceptability.
• Blends kept well for two weeks without significant physicochemical changes.

## 1 Introduction

Several studies have pointed out the importance of fruits and their association with reduced risk of developing chronic diseases. Frequent consumption of fruits and vegetables has the potential to contribute to good and healthy living, reduce the chance of cardiovascular disease, and have a protective role against diabetes and several non-communicable diseases (Bursac' Kovačević et al., 2020). The benefits of fruits are attributable to their natural compositions which include vitamins such as ascorbic acid and folic acid; minerals like potassium, calcium, iron; fibers, anti-oxidants. They also contain other bio-active compounds essential for human health which are responsible for their functional attributes (Martín-belloso et al.*,* 2016). These attributes in fruits make them qualify as functional foods. Functional foods are foods providing benefits beyond basic nutrition. They contain substances that have the potential to improve human physical and mental wellness as well as have the capacity to prevent nutrition-related diseases (Gibson and Williams, 2001).

Beverages are the most active functional food category as a result of their ease of dissolution of functional components and convenient method of consumption (Corbo et al., 2014). Functional beverages have become the recent interest of consumers as their awareness of fruit benefits increases and that of the food industry as it comes at the center of research and development (Cong et al., 2020). Fruit-based functional beverages are important sources of vitamin C, polyphenols, minerals, and dietary antioxidants that give protection against oxidative stress by increasing cell resistance to reactive oxygen species (ROS) in the system and are also of significant health benefit for the human body (Siro et al., 2008; Maria and Riccardo, 2020; Liu et al., 2021; Londoño et al., 2017). Recent advances made in functional beverages include optimization of ingredients for the formulation of novel functional beverages, exploitation of microorganisms to enhance functional benefits, the use of natural ingredients, valorization of fruit by-products as functional ingredients, use of non-digestible food ingredients which impact benefits to human, and application of non-thermal treatment to preserve their functional attributes (Granato et al., 2020; Sun-waterhouse, 2011; Nazir et al., 2019). To maximize the benefits from the base ingredients used in functional beverages, they have to be uniquely combined and synergistically formulated to address several modern health challenges such as eliminating reactive oxygen species which can improve the overall quality of life.

Under-utilized wild fruits particularly have a considerable amount of essential nutrients and compounds. Baobab (*Adansonia digitata*) and black-plum (*Syzygium cumini)* fruits are widely distributed in tropical Africa. They have recently attracted interest as functional fruits due to their exceptional compositions of bioactive compounds with the potential to contribute to food security as well as play vital roles in the nutrition of the people especially in the rural communities (Aworh and Charles, 2015; Akinnifesi et al., 2008).

Baobab is a naturally dry fruit that is high in pectin, dietary fiber, vitamins, and minerals. Baobab locally known in Tanzania as *Ubuyu*, in Nigeria as *Kuka* or *Ose* (Sidibe and Williams, 2002) has 10 times higher content in Vitamin C compared to orange (Kaboré et al., 2011), high in mineral content such as iron and potassium (Osman, 2004). Vitamin C (ascorbic acid) has a very good anti-oxidant potential as it increases cell resistance to oxidative stress as well as aid the absorption of iron, consequently a natural remedy for anemic conditions (Naidu, 2003). The high potassium content of baobab is important for regulating blood pressure, its minimal sodium content is also good for a healthy heart (Houston, 2011).

Black-plum locally called *Zambarau* also has a significant amount of bioactive compounds, which have functional benefits and it is used in the treatment of diabetes (Sharafeldin and Raza, 2015). Black-plum has been reported to have a high antioxidant capacity to reduce oxidative stress, inhibit growth and induce apoptosis of human breast cancer, have anti-inflammatory, anti-microbial, anti-bacterial, anti-fungal, free radical scavenging, gastro-protective and anti-diabetic properties (Tavares et al., 2016; Baliga et al., 2013; Pfuzia et al., 2013). Pineapple (*Ananas comosus*) is the most commonly consumed tropical fruit. It is considered a functional fruit as a result of its good source of dietary fiber and other phytochemicals such as carotenoids, flavonoids, phenolic acids, ascorbic acid required for good and healthy living (Ayala-Zavala et al., 2011).

The blending of fruits has previously been established to improve the nutritional and organoleptic qualities of the blends by synergistically contributing to human well-being when the benefits of all the fruits are combined (Shaheel et al., 2015). The blending of these fruits has the potential to combine their individual functional characteristics to combat malnutrition, especially micronutrient deficiency among the most vulnerable groups in Africa. This study aimed to evaluate the quality attributes and storage stability of the juice blends from pineapple, baobab fruit pulp, and black-plum fruits.

## 2 Materials and methods

### 2.1 Material collection and preparation

Baobab, black-plum, and pineapple fruits used in this study were obtained fresh from Arusha in the northern part of Tanzania in the year 2019 with an average annual temperature range of about 17–20 °C and average annual rainfall range of 800-1,200 mm. The indigenously grown pineapple variety (*smooth cayenne*) produced from the Tanga region (average annual temperature range of 30–32 °C and average annual rainfall range of 1,100–1,400 mm) in Tanzania was obtained from a local farmer's market. Wildly produced black-plum and baobab fruits were picked at commercial maturity in April 2019 harvest season from the Babati community. The local varieties of these fruits were identified at the nearby research center, Tengeru Agricultural Research Institute (TARI), Tengeru, Tanzania. Chemicals used were of analytical grade and purchased from Sigma-Aldrich and Merck. These include; Folin- Ciocalteau reagent, Trolox, Gallic acid, L-ascorbic acid standards, methanol, 1, 1- diphenyl-2-picrylhydrazyl (DPPH), and 2, 4- dinitrophenylhydrazine (DNPH).

The dry pulp of baobab fruits was manually separated from the pod and fiber using mortar and pestle, sieved, and blended into a fine powder. Blemish-free, thoroughly washed fully ripened black-plum fruits (indicated by the deep-purple color) were selected and treated according to the method of Rai et al. (2011), kept at the freezing temperature until used. Fully mature and ripened pineapple fruits (assessed at 12.3 °Brix) were selected and thoroughly washed under running tap water and peeled using stainless steel knives.

### 2.2 Extraction of juice and preparation of baobab-pineapple-black plum juice blends

The juice extraction of black-plum was done by the hot break pulping method of Lal et al. (1986) described by Sharma (2016). Water was added to whole fruits (1:1), heated at 60 °C for 5 min, homogenized, and double-filtered through a sieve to remove the seed and fibers. Baobab fruit pulp was minimally processed by adding the powder to potable water (1:10), properly homogenized, filtered using a muslin cloth and kept in a refrigerator prior to mixing. Pineapple juice was made using a juice extractor (Kenwood, JE680 series, China).

Separate juices of baobab, pineapple, and black-plum fruits were blended in different ratios, samples (F1–F7) while 100% baobab juice, 100% pineapple juice and 100% black-plum juice were extracted samples (F8–F10) without blending as shown in the mixing ratio given in Table 1. The juice blends were mixed in previously washed and sterilized bottles, pasteurized at 80 °C for 5 min (Tembo et al., 2017), cooled and immediately stored at refrigeration temperature (4 °C) in 50 mL bottles in the dark and changes in the physicochemical properties were evaluated at weekly intervals for 4 weeks.

**Table 1:** Blends of baobab, pineapple, and black-plum (BPB) fruit juices at different ratios (volume, %).

Fruit ​juices	F ​1	F2	F3	F4	F5	F6	F7	F8	F9	F10
Baobab	33.3	50	25	25	50	37.5	12.5	100	-	-
Pineapple	33.3	25	50	25	12.5	50	37.5	-	-	100
Black-plum	33.3	25	25	50	37.5	12.5	50	-	100	-

### 2.3 Analysis of the juice blends

#### 2.3.1 Blends physicochemical properties determination

Physicochemical properties such as pH, total titratable acidity (TTA), total soluble solids (TSS), and color of the juice blends were determined using the recommended standard method of analysis (AOAC, 2005) Method No: 2005.08. pH of the juices was measured using a digital pH meter (GMH Griesinger 3500 series, Germany) previously calibrated using 4 and 7 pH buffers. TTA of the juices was determined using a semi-titrator (Titronic Basic, UK). Brix was measured using a hand-held refractometer (Model Erma, Japan) of range 0–32 °Brix at 20 °C. The color of the samples was read using a spectrophotometer (MetaVue, X-rite, VS3200, Switzerland).

#### 2.3.2 Determination of vitamin C (ascorbic acid) content

The ascorbic acid content was determined using spectrophotometry method described by Kapur et al. (2012). Approximately, 3% bromine water (w/v) was added to 4 mL of centrifuged sample solution previously obtained from homogenizing 5 mL of juice with 25 mL of metaphosphoric acid - acetic acid solution, followed by the addition of 10% thiourea (w/v), and 1 mL of 2,4- dinitrophenylhydrazine. The mixture was incubated at 37 °C for 3 h and 85% H_2_SO_4_ (v/v) was added before reading the absorbance at 521 nm using a microplate reader (Synergy HTX, multi-mode reader, Chicago, USA). Ascorbic acid (100–1000 mg/L) standard was used and quantification was done using the standard curve.

#### 2.3.3 Total phenolic content (TPC) determination

Total phenolic contents of the juice samples were determined using the modified Folin-Ciocalteu spectrophotometry method described by Singleton et al. (1999). One milliliter of the juice samples was mixed with 9 mL of distilled water; 1.0 mL Folin–Ciocalteu reagent was added to the mixture and shaken. After 5 min, 10 mL of 20% NaCO_3_ (w/v) solution was added with mixing, the solution was immediately diluted to volume (25 mL) with distilled water and incubated at 23 °C for 90 min and absorbance was read at 750 nm. TPC was quantified by plotting the calibration curve using gallic acid (6.25–100 mg/L) as the standard and expressed as mg gallic acid equivalent (mg GAE/mL) of the sample.

#### 2.3.4 Determination of antioxidant capacity

##### 2.3.4.1 DPPH radical scavenging activity determination

DPPH radical scavenging activity of juice blends was determined using the method described by Shekhar and Anju (2014) with some modification. About 4 mL of 0.1 mM methanolic DPPH solution was added to 200 μL of the juice sample and left in the dark at room temperature for 30 min. Absorbance was read at 517 nm using a microplate reader and standard curve generated using Trolox standard (50–1000 μM).

##### 2.3.4.2 Ferric ion reducing antioxidant power (FRAP) assay

The reducing power of the juice was determined using FRAP assay following the method of Benzie and Strain (1999) with some modification. FRAP reagent was added to100 μL test samples and standards in 15 mL Falcon tubes, vortex mixed, and kept at 37 °C for 4 min. Absorbance was read at 593 nm using a microplate reader. Calibration curve was made using FeSO_4_.7H_2_O as the standard ranging from 100-1000 μM.

#### 2.3.5 Analysis of mineral content of juice blends

The mineral content of the juice blends was determined using (ICP) Inductively Coupled Plasma technique (iCAP7200 Duo, Thermo scientific, China) according to the method described by Akubor (2017).

### 2.4 Sensory evaluation

Sensory evaluation of the juice blends was conducted using 15 semi-trained panelists (trained for 3 h with blank labeled commercial juices) consisting of members of laboratory staff in NM-AIST, Tanzania using a nine-point hedonic scale. Consent of the panelists was obtained before performing the sensory tests, and ethical approval was not required for this evaluation since the juice blends were freshly prepared. Parameters such as taste, mouthfeel, flavor, color, and overall acceptability were evaluated. Water and cracker biscuits were provided for each panelist to clear and rinse their mouth after tasting each sample.

### 2.5 Statistical analysis

All determinations were done in triplicate and data were subjected to Analysis of Variance (ANOVA) at α = 0.05 and mean separation was done with Tukey's Studentized Range test where the model has a significant effect on the variability of the result, using SPSS statistical program (version 21.1 IBM SPSS).

## 3 Results and discussion

### 3.1 Physicochemical properties of baobab-pineapple-black plum juice blends

The physicochemical properties of the juice and juice blends of baobab, pineapple, and black-plum juices are shown in Table 2. The pH values of the samples ranged from 3.12 to 3.94. Although the pH of the blends indicated acidic values with a standard deviation not greater than 0.01, analysis of variance showed that there was a significant effect (P < 0.05) of the model on the variability that is shown on the pH. The low pH of baobab juice contributed to the lower pH of all juice blends and may be attributed to the high concentration of organic acid present, predominantly citric acid (Tembo et al., 2017). The pH value obtained in this study was comparable to the pH of 3.15 reported for *Adansonia* (Adam et al., 2016), 3.79 for pineapple juice (Ngereza and Pawelzik, 2016), and 3.84–3.92 for pineapple-carrot-orange juice blends (Hossain et al., 2016). Low pH is associated with the microbial stability of food as the lower the pH, the more microbiologically stable the fruit juice (Nwachukwu and Ezeigbo, 2013).

**Table 2:** Physicochemical characteristics of juice blends from Baobab (*Adansonia digitata)*, Pineapple (*Ananas comosus*) and Black-plum (S*yzygium cumini)* fruits.

Samples	pH	TSS (°Brix)	TTA (% citric acid)	Color		
				L∗	a∗	b∗
F1	3.47 ± 0.01^c^	7.80 ± 0.14^c^	0.35 ± 0.01^c^	24.40 ± 0.30^c^	14.33 ± 0.25^a^	14.15 ± 0.49^b^
F2	3.30 ± 0.01^c^	7.30 ± 0.07^c^	0.41 ± 0.00^b^	29.67 ± 0.05^c^	11.59 ± 0.01^b^	12.95 ± 0.08^b^
F3	3.53 ± 0.01^b^	8.87 ± 0.04^b^	0.30 ± 0.00^c^	29.65 ± 0.18^c^	9.32 ± 0.09^c^	12.38 ± 0.24^b^
F4	3.58 ± 0.01^b^	7.83 ± 0.04^b^	0.34 ± 0.00^c^	22.42 ± 0.05^c^	14.23 ± 0.09^a^	9.59 ± 0.16^c^
F5	3.34 ± 0.01^c^	6.35 ± 0.07^d^	0.40 ± 0.00^b^	25.83 ± 0.09^c^	14.22 ± 0.05^a^	11.28 ± 0.15^b^
F6	3.44 ± 0.01^c^	8.05 ± 0.07^b^	0.40 ± 0.00^b^	22.76 ± 0.08^c^	15.79 ± 0.02∗^c^	9.80 ± 0.13^c^
F7	3.66 ± 0.01^b^	8.23 ± 0.04^b^	0.30 ± 0.00^c^	24.15 ± 0.12^c^	11.85 ± 0.06^b^	12.67 ± 0.29^b^
F8 ​100%Ba	3.12 ± 0.00^d^	4.83 ± 0.04^e^	0.62 ± 0.00^a^	41.22 ± 0.04^a^	4.31 ± 0.06^d^	25.37 ± 0.09^a^
F9 ​100%Bp	3.88 ± 0.01^a^	6.05 ± 0.07^d^	0.22 ± 0.00^d^	14.08 ± 0.11^d^	15.240.08^a^	6.42 ± 0.33^c^
F10 ​100% ​P	3.94 ± 0.02^a^	12.3 ± 0.07^a^	0.30 ± 0.01^c^	37.92 ± 0.09^b^	1.28 ± 0.02^d^	19.74 ± 0.20^a^

Ba**=**Baobab juice, P**=**Pineapple juice, Bp = Black-plum juice, TSS = Total Soluble Solids, TTA = Titratable acidity.

F1 = 33.3%Ba: 33.3%P: 33.3%Bp; F2 = 50%Ba: 25%P: 25%Bp; F3 = 25%Ba: 50%P: 25%Bp: F4 = 25%Ba: 25%P:50%Bp; F5 = 50%Ba:12.5%P:37.5%Bp; F 6 = 37.5%Ba: 50%P: 12.5%Bp; F7 = 12.5%Ba: 37.5%P:50%Bp; F 8 = 100% Baobab juice; F 9 = 100% Black-plum juice; F 10 = 100% Pineapple juice.

Values are means of triplicates (n = 3) ± standard deviations. Mean with different letter superscripts in the same column are significantly different (P < 0.05).

Total soluble solid (TSS) is a measure of soluble sugars in juices. The TSS in the samples ranged between 4.83 (for 100% baobab juice) and 12.3 °Brix (for 100% pineapple juice) (Table 2). The effect of blending and type of fruits significantly affected the Brix values of the blends and pure juices. The TSS values (6.35–8.87) for the blends fall between the ranges for the pure juice as expected. The lower TSS in the juice blends could be attributable to baobab juice with the least amount of sugar content of the pure juices. Pineapple juice has the highest Brix of (12.3 °B), and this may be attributed to the high content of sucrose naturally present in it (Ngereza and Pawelzik, 2016; Akubor, 2017). Juice blend with high pineapple juice (F3, F6, and F7) had the correspondingly high TSS. A similar result was reported by Hossain et al. (2016) and Begam et al. (2018).

The titratable acidity of the samples ranged between 0.22% and 0.62% (Table 2). Baobab juice had the least pH of 3.12, and the highest acidity of 0.62%. The expectation was that low pH should correspond to high acidity (Bamidele and Fasogbon, 2017), which the results from this study confirm. The predominant organic acid (citric acid) present in baobab may be responsible for this high acidity value. The effect of baobab juice's high acidity was observed in samples F2, F5, and F6.

Color is an important quality parameter of beverage contributing to organoleptic properties and subsequent consumer acceptability. The color parameters of the samples presented in Table 2 show the lightness values (L∗) range from 14.08 to 41.22. Sample F9, 100% black-plum juice had the lowest (L∗ = 14.08) as expected from the dark pigmentation of the fruit, while sample F8, 100% baobab juice had the highest (L∗ = 41.22). The highest redness values (a∗) was found in sample F9 (a∗ = 15.24) and lowest in sample F10 (a∗ = 1.28). The blending of the fruit juices resulted in a significant (P < 0.05) change in colorimetric values in all the samples. The significantly high value of (a∗) in juice blends (F1, F4, and F5) may have resulted from high polyphenol content especially anthocyanin compound in black-plum juice. Yellowness (b∗) values of the juice samples ranged from 6.42 to 19.74 with sample F10 having the highest. A similar result was observed by Kamarul and Shamsudin (2016) for pineapple and mango juice blends that the color of the juice samples varies with different juice concentrations.

### *3.2* Phytochemical properties of baobab-pineapple-black plum (BPB) juice blends

**Figure 1:**
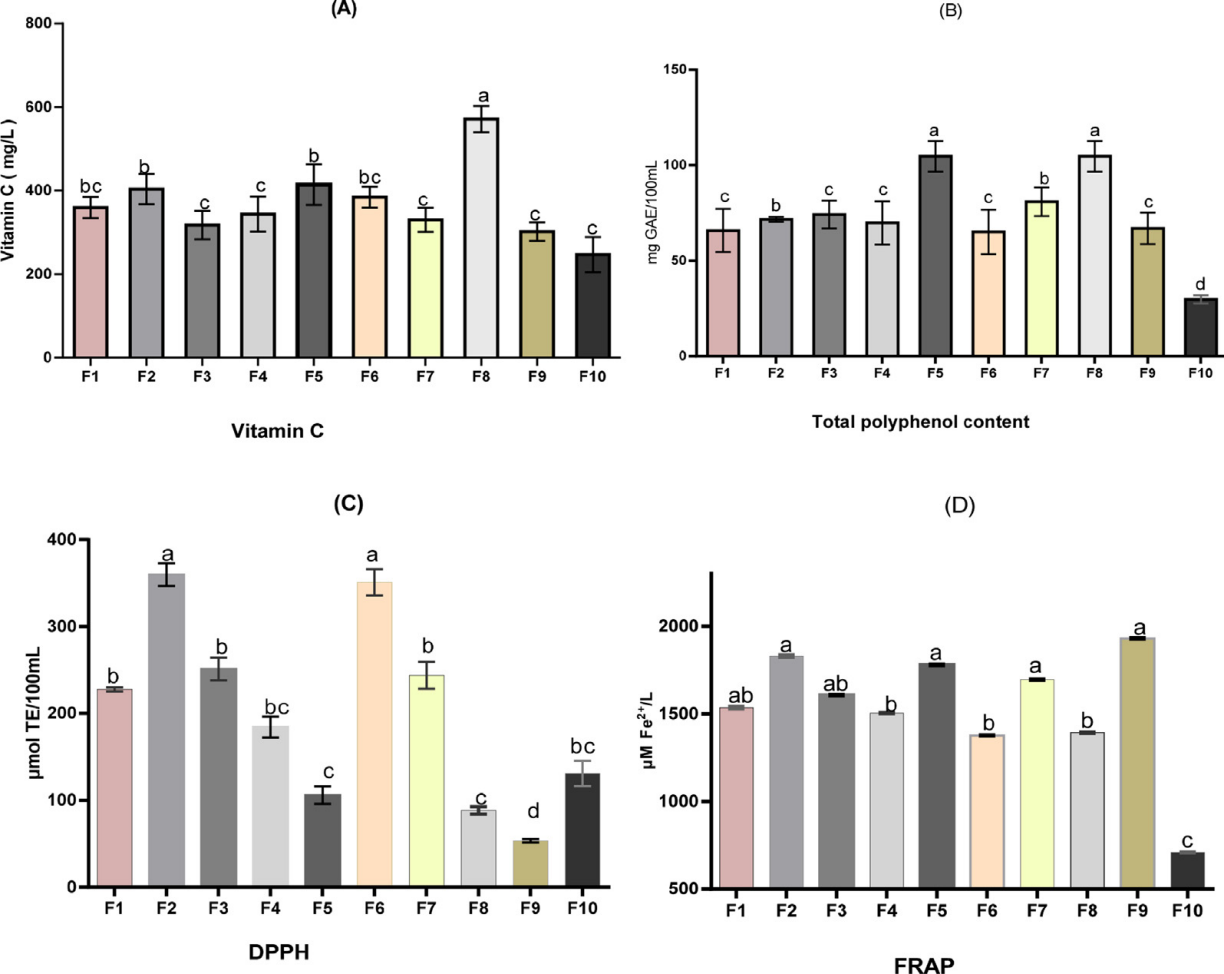
The vitamin C content (A), total phenolic content (B), DPPH radical scavenging activity (C), and Ferric ion reducing antioxidant power (D) of juice blends from Baobab, Pineapple, and Black-plum fruits. Ba**=**Baobab juice, P**=**Pineapple juice, Bp = Black-plum juice, FRAP = Ferric ion reducing antioxidant power, DPPH = 1,1-diphenyl-2-picrylhydrazyl. F1 = 33.3%Ba: 33.3%P: 33.3%Bp; F2 = 50%Ba: 25%P: 25%Bp; F3 = 25%Ba: 50%P: 25%Bp: F4 = 25%Ba: 25%P: 50%Bp; F5 = 50%Ba: 12.5%P: 37.5%Bp; F 6 = 37.5%Ba: 50%P: 12.5%Bp; F7 = 12.5%Ba: 37.5%P: 50%Bp; F 8 = 100% Baobab juice: F 9 = 100% Black-plum juice F10 = 100% Pineapple juice. Values are means of triplicates (n = 3) ± standard deviations. Significant differences between formulations (F1 to F10) are indicated by different letters (P < 0.05).

Figure 1 presents the phytochemical properties of the baobab-pineapple-black plum juice blends. Vitamin C content, total polyphenol content, and antioxidant capacities were determined in this study. Figure 1A showed the vitamin C content of the juice samples with 100% baobab (sample F8) having the highest (571.52 mg/L) value. The significantly high (P < 0.05) vitamin C content of the blends could be attributed to their baobab content since baobab fruit pulp is reported to be a good source of vitamin C (Osman, 2004; USDA, 2018). Sample F8 ascorbic acid content differs from sample F9 and sample F10 at P < 0.05. This confirms that black-plum and pineapple juices are also fair sources of vitamin C (Bukya and Madane, 2018; Masamba and Mndalira, 2013). The synergistic combination of all vitamin C in the individual juices contributed to the high vitamin C observed in the blends with blends having a lesser amount of baobab juice having lower vitamin C content. Although citrus fruits are previously recognized as important sources of vitamin C (Rudge et al., 2012; Giuffrè et al., 2017) having beneficial effects on humans, these findings established other underutilized tropical fruits such as baobab and black-plum containing this valuable vitamin. The amount of vitamin C observed in the juice blend samples could cover about 60% of the recommended dietary allowances (RDA) based on the recommendation that dietary intake of 90–100 mg ascorbic acid/day could reduce the risk of non-communicable diseases (Carr and Frei, 1999). A similar trend was observed in the total polyphenol content (TPC) values as in vitamin C content. The TPC contents of all the samples ranged from 29.81 to 104.65 mg GAE/100 mL with the highest value found in 100% baobab juice (sample F8) and sample F5. Vitamin C is an important nutrient that performs several biological functions in the human body such as preventing free radical damage to DNA and providing support to the immune system (Naidu, 2003).

The reducing potential and DPPH radical scavenging capacity of juice samples are shown in Figure 1(C-D). Black-plum juice showed the highest ability to reduce Fe^3+^ to Fe^2+^(1929 μMFe^2+^/L) followed by baobab juice (1391.81 μMFe^2+^/L). Among the juice blend, the reducing power is significantly high, showing the capability of the juice blends to donate electrons in order to terminate chain reactions resulting from free radical oxidation. The presence of free radical scavenging ability in the juice samples was significant thus the juices demonstrate high anti-oxidant properties despite their variations. There was a significant difference (P < 0.05) in the DPPH radical scavenging ability of the samples, with samples F2 and F6 having the highest values. Baobab and black-plum have been reported to have high antioxidant properties and so can prevent the risk of disease such as cancer, diabetes resulting from the free radical-induced oxidative reaction (Braca et al., 2018; Baliga et al., 2013).

The synergetic effect resulting from the combined relatively high scavenging ability, high reducing potential, high ascorbic acid content, high polyphenol contents of the juice blends observed in this study, translates into increased antioxidant capacity thus its potential benefits of reducing free radicals when consumed in sufficient amount. Similarly, Aderinola and Abaire (2019) and Olagunju and Sandewa (2018) reported increased antioxidant content of cucumber and carrot juice; soursop juice and milk respectively.

### 3.3 Mineral content of juice blends

The results of the mineral contents of the samples are shown in Table 3. The juice blends showed high content of all the minerals tested, particularly magnesium, potassium, calcium, and phosphorus. The concentration of calcium was high (57–153 mg/L) compared to the other dietary minerals in the samples. Other elements varied between (71–130 mg/L) for magnesium, potassium (22–32 mg/L), phosphorus (4–43 mg/L), and sodium (0.79–1.97 mg/L). Similar high calcium content (80.3 mg/kg) was reported for orange juice (Simpkins et al., 2000) and 282 mg/kg for baobab fruit pulp (Adam et al., 2016). Calcium is an important mineral for muscles and bone health (Pravina et al., 2013). This makes these blends a good source of calcium supplements for lactating women and children. Potassium is also an essential component of body cells and fluid while low sodium is ideal for a healthy heart.

**Table 3:** Mineral content determination for the juice blends (mg/L).

Samples	Magnesium	Potassium	Calcium	Iron	Zinc	Phosphorus	Sodium
F1	80.20 ± 10.65^b^	25.11 ± 1.83^b^	70.35 ± 10.59^bc^	0.04 ± 3.73^c^	1.15 ± 0.01^b^	24.11 ± 1.11^b^	1.97 ± 0.02^c^
F2	73.50 ± 9.19^c^	24.08 ± 2.31^b^	77.23 ± 7.41^bc^	0.04 ± 6.30^c^	1.11 ± 0.01^b^	17.12 ± 0.15^c^	1.56 ± 0.01^c^
F3	98.23 ± 10.79^b^	29.45 ± 3.81^ab^	72.36 ± 10.58^bc^	0.04 ± 0.45^c^	1.15 ± 0.02^b^	25.15 ± 1.01^b^	2.23 ± 0.01^b^
F4	85.15 ± 10.02^b^	26.04 ± 1.45^b^	67.08 ± 9.62^c^	0.05 ± 8.51^c^	0.49 ± 0.01^c^	28.01 ± 1.01^b^	1.51 ± 0.02^c^
F5	73.12 ± 9.57^c^	32.54 ± 2.82^a^	89.45 ± 10.05^b^	0.05 ± 7.29^c^	1.08 ± 0.03^b^	19.24 ± 0.02^c^	1.32 ± 0.20^c^
F6	76.19 ± 6.46^c^	25.12 ± 1.46^b^	57.03 ± 5.84^c^	0.01 ± 3.02^c^	1.08 ± 0.02^b^	27.21 ± 0.09^b^	1.61 ± 0.05^c^
F7	92.13 ± 5.59^b^	27.49 ± 2.40^ab^	119.02 ± 4.87^a^	0.07 ± 2.15^c^	0.51 ± 0.02^c^	30.32 ± 0.03^b^	1.36 ± 0.02^c^
F8 ​100%Ba	71.07 ± 3.18^c^	22.02 ± 2.54^c^	153.42 ± 2.21^a^	0.18 ± 1.69^b^	1.01 ± 0.01^b^	4.00 ± 0.04^d^	0.79 ± 0.01^d^
F9 ​100%Bp	83.14 ± 1.73^b^	28.31 ± 1.66^ab^	19.24 ± 0.03^d^	0.09 ± 4.84^c^	1.22 ± 0.02^a^	43.46 ± 2.03^a^	1.53 ± 0.03^c^
F10 ​100% ​P	130.23 ± 5.53^a^	30.06 ± 2.20^a^	65.09 ± 6.11^c^	0.32 ± 1.02^a^	1.21 ± 0.02^a^	28.09 ± 2.01^b^	3.60 ± 0.20^a^

Ba**=**Baobab juice, P**=**Pineapple juice, Bp = Black-plum juice.

F1 = 33.3%Ba: 33.3%P: 33.3%Bp; F2 = 50%Ba: 25%P: 25%Bp; F3 = 25%Ba: 50%P: 25%Bp: F4 = 25%Ba: 25%P:50%Bp; F5 = 50%Ba:12.5%P:37.5%Bp; F 6 = 37.5%Ba: 50%P: 12.5%Bp; F7 = 12.5%Ba: 37.5%P:50%Bp; F 8 = 100% Baobab juice; F 9 = 100% Black-plum juice; F 10 = 100% Pineapple juice.

Values are means of triplicates (n = 3) ± standard deviations. Means values with the same letter in a column are not significantly different (P > 0.05).

### 3.4 Storage studies of the juice blend

**Figure 2:**
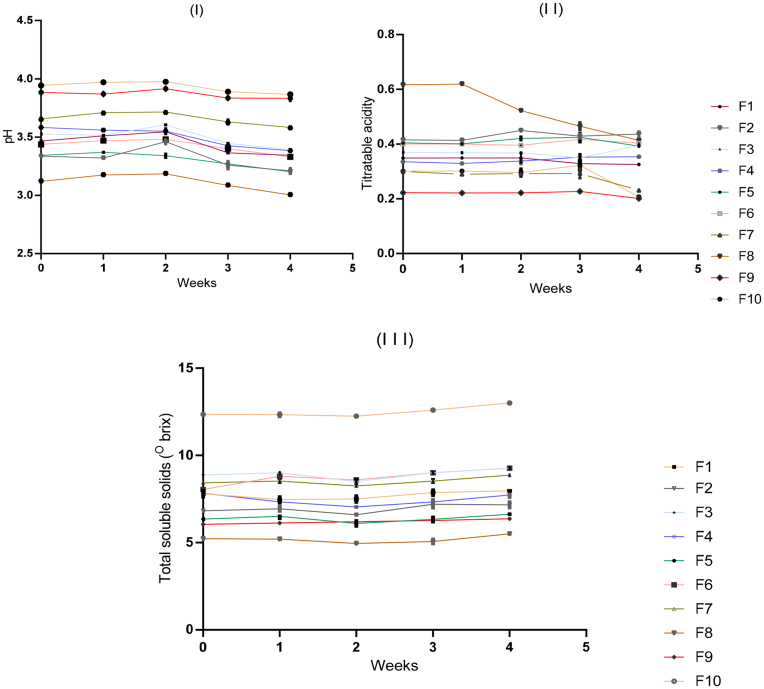
Storage effects on physicochemical characteristics of juice blends from baobab, pineapple and black-plum fruits; (I) pH, (II) Titratable acidity, (III) Total soluble solids (^o^ brix). F1 = 33.3%Ba: 33.3%P: 33.3%Bp; F2 = 50%Ba: 25%P: 25%Bp; F3 = 25%Ba: 50%P: 25%Bp; F4 = 25%Ba: 25%P: 50%Bp; F5 = 50%Ba: 12.5%P: 37.5%Bp; F 6 = 37.5%Ba: 50%P: 12.5%Bp; F7 = 12.5%Ba: 37.5%P: 50%Bp; F 8 = 100% Baobab juice; F 9 = 100% Black-plum juice; F10 = 100% Pineapple juice. Values are means of triplicates (n = 3) ± standard errors.

Figure 2 shows the effect of storage at refrigeration temperature (4 °C) for 4 weeks on pH, acidity, and TSS of baobab-pineapple-black plum juice blends. There was no significant change in the pH of the juice blends at the first 2 weeks of storage but a slight decrease in pH occurred after week 2 until week 4 of storage. This may be due to biochemical reactions occurring within the juice samples. The relatively unchanged low pH (3.01–3.86) observed in all the samples is an indication of shelf-stability at 2 weeks under refrigeration temperature even though the color became less intense at the end of the storage studies. This observation was supported by the report of Nwachukwu and Ezeigbo (2013) that pasteurized fruit juices at acidic pH under refrigeration temperature are less susceptible to contamination and growth of microorganisms. Moreso, the acidity of the juice blends showed no significant change in the first 2 weeks of the storage period except for a slight decrease towards the end of week 4. The presence of natural antioxidants and organic acids with pasteurization and low temperature are important useful factors in extending the shelf-life of fruit juices. There was a slight increase in the TSS towards the end of the storage period (Fig. 2III). This could be attributed to the hydrolysis of polysaccharides to sugar. Bhardwaj and Pandey (2011) also reported an increase in TSS value as storage time and temperature increased. Therefore, it can be concluded from this study that juice blends of baobab, pineapple, and black-plum were kept well without significant physicochemical changes at low temperatures (4 °C) for 2 weeks even as the color become less intense.

### 3.5 Sensory properties of baobab-pineapple-black plum (BPB) juice blends

The results of sensory properties such as taste, mouthfeel, flavor, color, and overall acceptability of the juice samples are shown in Table 4. Sample F8 was rated lowest for all the evaluated parameters except for the color that was rated high. This may be due to the high acidity of the juice resulting in its astringency and sourness. This sample coincidentally has the highest value of the phytonutrients tested (Figure 1). A similar low rating for samples with better antioxidant properties was observed in the blend of an equal volume of carrot and cucumber juice (Aderinola, 2019). Sample F10 rated highest for taste, mouthfeel, flavor, color, and overall acceptability. This may be due to the high sugar content reflected in the high Brix value of the sample (Table 2) and the familiar color of the juice to the consumers (Akubor, 2017). Sample F9 rated high only for color but low for other parameters. This may be due to the attractive color (light purple) resulting from the high anthocyanin content of the juice. The low overall acceptability of this juice may be due to the pungent smell and watery taste resulting from low Brix value. Samples F1, F3, F4, F6, and F7 were rated high for overall acceptability among the juice blends. This may be due to the high ratio of pineapple juice above 25% in the blends and baobab juice, not more than 25%, especially in samples F3 and F7. This implies that blending baobab with pineapple and black plum can complement and improve consumers' acceptability. Although there was no significant difference (P > 0.05) in the ratings among the juice blends, sample F5 showed better phytonutrient properties but was poorly rated by consumers. Consumer acceptance could be improved upon in the final formulation for a ready-to-drink functional beverage.

**Table 4:** Sensory attributes of the juice blends from Baobab, Pineapple and Black-plum fruits.

Samples	Taste	Mouthfeel	Flavor	Color	Overall acceptability
F1	6.93 ± 1.34^b^	7.00 ± 1.36^b^	6.87 ± 0.99^bc^	7.40 ± 1.18^b^	6.93 ± 1.22^bc^
F2	6.07 ± 1.39^c^	6.00 ± 1.00^c^	6.33 ± 0.90^c^	7.20 ± 0.68^b^	6.20 ± 1.08^c^
F3	7.47 ± 0.99^b^	7.53 ± 0.99^ab^	7.47 ± 0.92^b^	7.53 ± 0.99^a^	7.47 ± 0.95^b^
F4	7.07 ± 1.22^b^	7.00 ± 0.85^b^	6.80 ± 1.04^bc^	7.67 ± 0.90^a^	7.13 ± 0.92^bc^
F5	6.00 ± 1.31^c^	6.33 ± 1.05^c^	6.13 ± 0.99^c^	6.93 ± 1.49^c^	6.40 ± 0.74^c^
F6	7.20 ± 1.27^b^	7.33 ± 1.11^b^	7.07 ± 1.16^b^	7.07 ± 1.03^ab^	7.07 ± 1.10^bc^
F7	7.53 ± 0.92^b^	7.20 ± 1.08^b^	7.07 ± 1.39^b^	7.60 ± 0.74^a^	7.60 ± 0.83^b^
F8 ​100%Ba	4.00 ± 1.89^d^	4.33 ± 2.19^d^	5.02 ± 2.04^d^	6.80 ± 1.86^c^	4.80 ± 0.19^d^
F9 ​100%Bp	5.00 ± 2.45^d^	5.07 ± 2.34^d^	4.93 ± 2.31^d^	7.07 ± 1.75^ab^	5.33 ± 2.19^d^
F10 ​100% ​P	8.13 ± 0.99^a^	8.13 ± 0.74^a^	8.33 ± 0.82^a^	7.60 ± 0.91^a^	8.27 ± 0.46^a^

Ba=Baobab juice, P=Pineapple juice, Bp = Black-plum juice.

F1 = 33.3%Ba: 33.3%P: 33.3%Bp; F2 = 50%Ba: 25%P: 25%Bp; F3 = 25%Ba: 50%P: 25%Bp: F4 = 25%Ba: 25%P:50%Bp; F5 = 50%Ba:12.5%P:37.5%Bp; F 6 = 37.5%Ba: 50%P: 12.5%Bp.

F7 = 12.5%Ba: 37.5%P:50%Bp); F 8 = 100% Baobab juice; F 9 = 100% Black-plum juice; F 10 = 100% Pineapple juice.

Values are means of triplicates (n = 3) ± standard deviations. Mean with different letter superscripts in the same column are significantly different (P < 0.05).

## 4 Conclusions

Baobab-pineapple-black plum juice blends showed a high amount of micronutrients and phytochemicals that establish their potential as functional beverages vital in disease prevention and health promotion. The blending of the fruit juices improved the sensory attributes and subsequently consumers’ acceptance. The present study revealed that pasteurized baobab-pineapple-black plum juice blends kept well at a low temperature until 2 weeks with no significant change in physicochemical quality attributes. Juice blends of baobab, pineapple, black-plum fruits synergistically increased the anti-oxidant potential and mineral contents. This study provides important information highlighting the quality attributes of blends of baobab, pineapple, and black plum juices which can contribute to reducing micronutrient deficiency and improving the nutritional status of people in the rural communities in sub-Saharan Africa. Additionally, it provides a guide to the food industry in the development of new functional beverages improving the utilization and economic value of these fruits and becoming a source of income generation for the producers and retailers.

## Declarations

### Author contribution statement

Tawakalt O. Adedokun: Conceived and designed the experiments; Performed the experiments; Analyzed and interpreted the data; Wrote the paper.

Athanasia Matemu: Conceived and designed the experiments.

Otmar Höglinger: Performed the experiments; Contributed reagents, materials, analysis tools or data.

Erasto Mlyuka, Akinbode Adedeji: Conceived and designed the experiments; Analyzed and interpreted the data.

### Funding statement

This work was supported by the Centre for Research, Agricultural Advancement, Teaching Excellence and Sustainability (CREATES), and Nelson Mandela African Institution of Science and Technology (NM-AIST), Arusha, Tanzania, through the fund provided by the World Bank.

### Data availability statement

Data will be made available on request.

### Declaration of interests statement

The authors declare no conflict of interest.

### Additional information

No additional information is available for this paper.
